# Precision delivery of liquid therapy into the arterial wall for the treatment of peripheral arterial disease

**DOI:** 10.1038/s41598-021-98063-z

**Published:** 2021-09-21

**Authors:** Marzieh K. Atigh, Emily Goel, Megan Erwin, Ricky Greer, Jacques Ohayon, Roderic I. Pettigrew, Saami K. Yazdani

**Affiliations:** 1 Department of Mechanical Engineering, University of South Alabama, Mobile, AL 36688 USA; 2 Savoie Mont-Blanc University, Polytech Annecy-Chambéry, Le Bourget du Lac, France; 3 Laboratory TIMC-IMAG, CNRS, UMR 5525, Grenoble-Alpes University, Grenoble, France; 4 Texas A&M University and Houston Methodist Hospital, Engineering Medicine (EnMed), Houston, TX USA; 5 Department of Engineering, Wake Forest University, Winston-Salem, NC 27101 USA

**Keywords:** Biomedical engineering, Preclinical research, Interventional cardiology

## Abstract

Perfusion catheters have recently emerged as a novel approach to deliver liquid anti-proliferative agents into flow obstructed arterial segments. The purpose of this study was to determine the impact of luminal delivery pressure on liquid drug penetration into the vessel wall. An ex vivo model using harvested porcine carotid arteries and a two-dimensional computational model were utilized to determine the impact of delivery pressure of liquid therapy into the arterial wall. A pig peripheral injury model determined the impact of intra-luminal delivery pressure on drug retention. Ex vivo results demonstrated that depth of fluid penetration varies from 6.93 ± 1.90% at 0 atm to 27.75 ± 6.61% penetration of the medial layer at 0.4 atm. Computational results had similar outcomes, as penetration varied between 4.4% and 22.84%. The in vivo results demonstrated significant increase in drug delivery to the arterial tissue at 0.4 atm versus 0.1 atm at 1 h (23.43 ± 13.59 ng/mg vs. 2.49 ± 1.81 ng/mg, p = 0.026) and 7 days (0.50 ± 0.39 ng/mg vs. 0.018 ± 0.023 ng/mg, p = 0.0496). The result of this study provides an innovative strategic and technical approach to enable targeted liquid therapy.

## Introduction

In the United States, treatment of peripheral artery disease (PAD) costs roughly $21 billion annually and affects over 8 million people^1^. Caused by an inflammatory process that results in atherosclerosis, plaque build-up narrows and restricts blood flow causing pain, mobility loss and poor wound healing. The various approaches to treat PAD include bypass surgery, balloon angiography, and stent placement, although more recently, there has a been a surge in the number of new devices to treat patients with PAD^2–5^. Percutaneous intervention including drug eluting stent (DES), balloon angioplasty or drug coated balloon (DCB), is preferred over bypass surgery based on lower morbidity and mortality rates and shorter in-hospital stays^6,7^.

The strategy of DES and DCB are to deliver and deposit a high concentration of anti-proliferative agents, such as paclitaxel and sirolimus, onto the luminal surface^8,9^. The potential problem of this approach is poor re-endothelialization of the luminal surface, delayed vascular healing and potential for thrombosis^10,11^. DCBs, which are an angioplasty balloon coated with an anti-proliferative drug in combination with an excipient (drug carrier), were developed as an alternative treatment method to locally delivery anti-proliferative agents^12^. They have shown to be more successful than DES in the treatment of PAD^13^. As the outer surface of the coated balloon comes in contact with the arterial wall, the drug and excipient are transferred onto the luminal surface of the artery. Paclitaxel has been the most widely used anti-proliferative drug used in all current FDA-approved DCBs^14^.

Perfusion catheters are a new class of device that can locally administer drugs in liquid form within arteries to treat vascular disease^15–17^. The perfusion catheter is the ultimate leave nothing behind strategy for treating occlusive arterial disease. Unlike DES and DCBs, which deposit therapeutics on the luminal surface, with the unintended consequence of inhibiting re-endothelialization, perfusion catheters deliver drug directly into the vessel wall. In DCBs, drug transfer from the balloon surface to the vessel wall ranges from 10 to 20%^18,19^. Perfusion catheters deliver the therapeutic by isolating a region of the artery, halting blood flow, and filling the isolated luminal space with the liquid drug solution. The drug solution can then be pressurized to drive the solution into the arterial wall. This novel approach can provide a critical difference in the effectiveness of modern intervention treatment of cardiovascular disease. The local liquid delivery approach can deliver therapeutic drug into any vessel wall regardless of the cross‐sectional shape. In DCB however, successful delivery of drug coated on the surface of the balloon is highly dependent on the cross‐sectional shape of the vessel, with a preference toward a circular cross‐sectional area to maximize balloon‐to‐artery contact area. The use of a perfusion catheter also eliminates the potential loss of drug in transit/tracking and provides the opportunity to treat multiple lesions with a single device per patient.

In this study, we investigated the factors influencing the penetration of liquid therapy into the artery wall. Specifically, we evaluated the effects of variations in luminal delivery pressure on liquid drug penetration. This was accomplished with the use of an ex vivo experimental model using fluorescently labeled drug (Flutax-1) to visualize penetration into the artery wall. Additionally, a two-dimensional, pressure-dependent model of an arterial wall and a finite-element solution under similar delivery conditions was utilized. Finally, differences in delivery parameters on drug retention was evaluated in vivo using a porcine injury model. The results obtained in the study give insight into how luminal delivery pressure may be selected to improve penetration in local liquid delivery approaches.

## Results

### Ex vivo experiments

**Figure 1:**
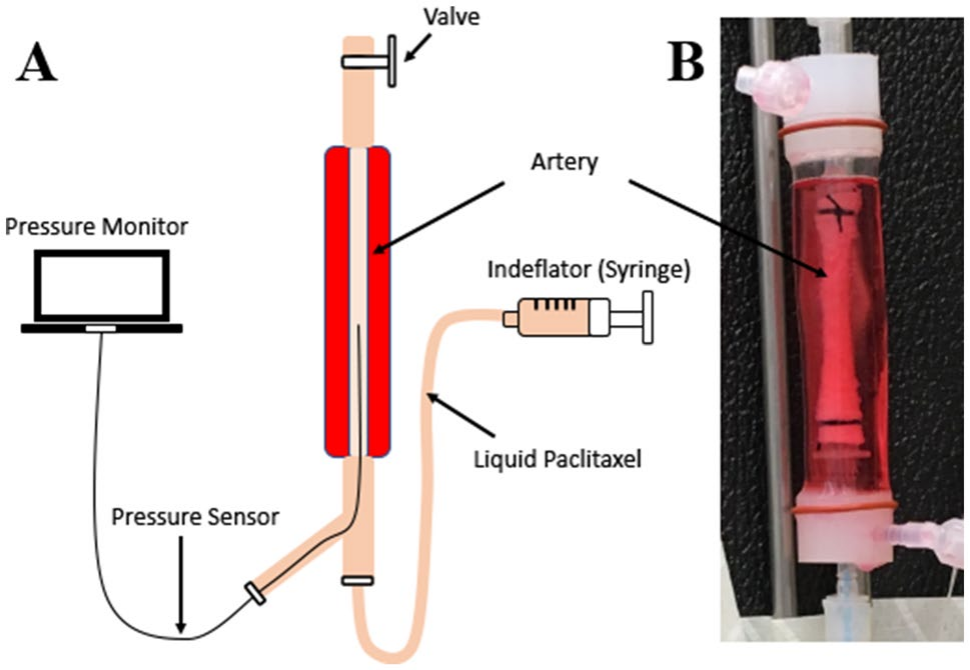
Ex vivo experimental setup. (**A**) Schematic diagram of the ex vivo setup. A pressure sensor inserted into the lumen continuously monitored pressure during the delivery of the liquid solution. (**B**) Representative image of a harvested porcine carotid artery housed in the bioreactor apparatus.

**Figure 2:**
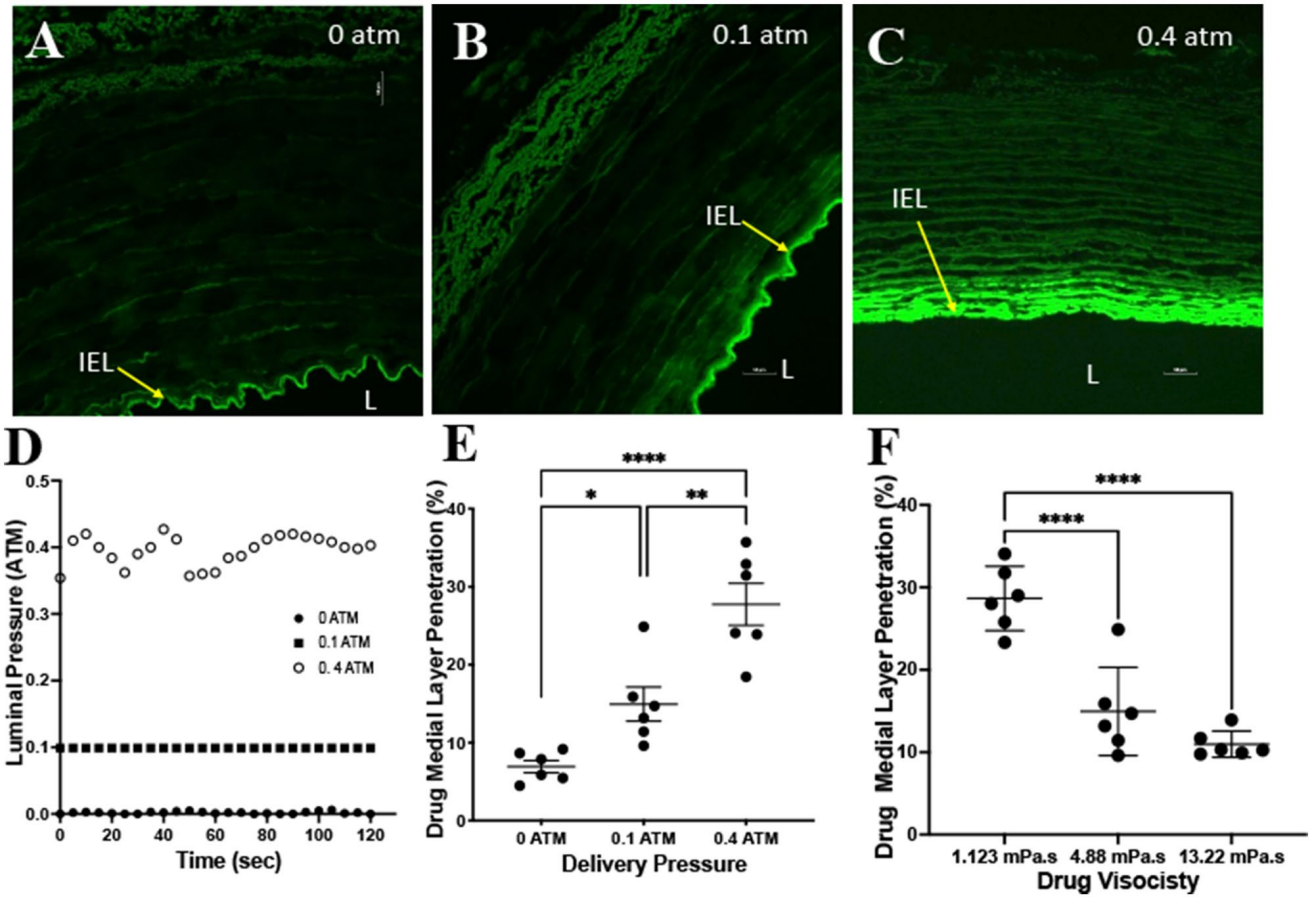
Ex vivo results of varying pressures and viscosities. (**A**–**C**) Representative confocal images demonstrating the penetration of the fluorescent drug at luminal pressures of 0 atm, 0.1 atm and 0.4 atm. Yellow arrow denotes the internal elastic lamina (IEL) and L represents the lumen of the artery. (**D**) Representative luminal pressure measurements during the two-minute drug delivery. (**E**) Drug medial layer penetration comparisons between the varying luminal pressures. (**F**) Drug medial layer penetration comparisons between varying liquid drug viscosities. *P < 0.05, **P < 0.01, ****P < 0.0001, *n* = 6.

A representative image of the ex vivo system is shown in Fig. 1. Experiments ranging in luminal pressures from 0 to 0.4 atm and a dynamic viscosity of 4.88 mPa s were performed. The measured average pressure for the 0 atm group was 0.0019 ± 0.0018 atm, 0.099 ± 0.00003 for the 0.1 atm group and 0.396 ± 0.022 for the 0.4 atm group (Fig. 2). Confocal microscopy imaging demonstrated uniform circumferential delivery of the Flutax-1into the arterial wall. As expected, penetration into the medial layer, represented as a percentage of penetration depth normalized wall thickness, at a zero pressure was minimal at 6.93 ± 1.90%. At 0.1 atm, penetration significantly increased to 14.95 ± 5.36%, as compared to 0 atm (p = 0.0366). At 0.4 atm, penetration increased and was significantly greater than the penetration at zero pressure (0.4 atm: 27.75 ± 6.61% vs 0 atm: 6.93 ± 1.90%, p = 0.0001) and 0.1 atm (0.4 atm: 28.57 ± 10.13% vs 0.1 atm: 14.95 ± 5.36%, p = 0.0014).

To determine the impact of viscosity on liquid drug penetration, we selected three different viscosity measurements (1.12 mPa s, 4.88 mPa s, 13.22 mPa s) all delivered at a luminal pressure of 0.1 atm. The results demonstrated a significant difference in drug penetration with a viscosity of 1.12 mPa s as compared to 4.88 mPa s (1.12 mPa s: 28.66 ± 3.90% vs 4.88 mPa s: 14.95 ± 5.36%, p = 0.0001) and as compared to 13.22 mPa s (1.12 mPa s: 28.66 ± 3.90% vs 13.22 mPa s: 10.97 ± 1.59%, p = 0.0001). There was no significant difference in drug medial penetration between the 4.88 mPa s and 13.22 mPa s at a delivery pressure of 0.1 atm (p = 0.22).

### Mathematical modeling

**Figure 3:**
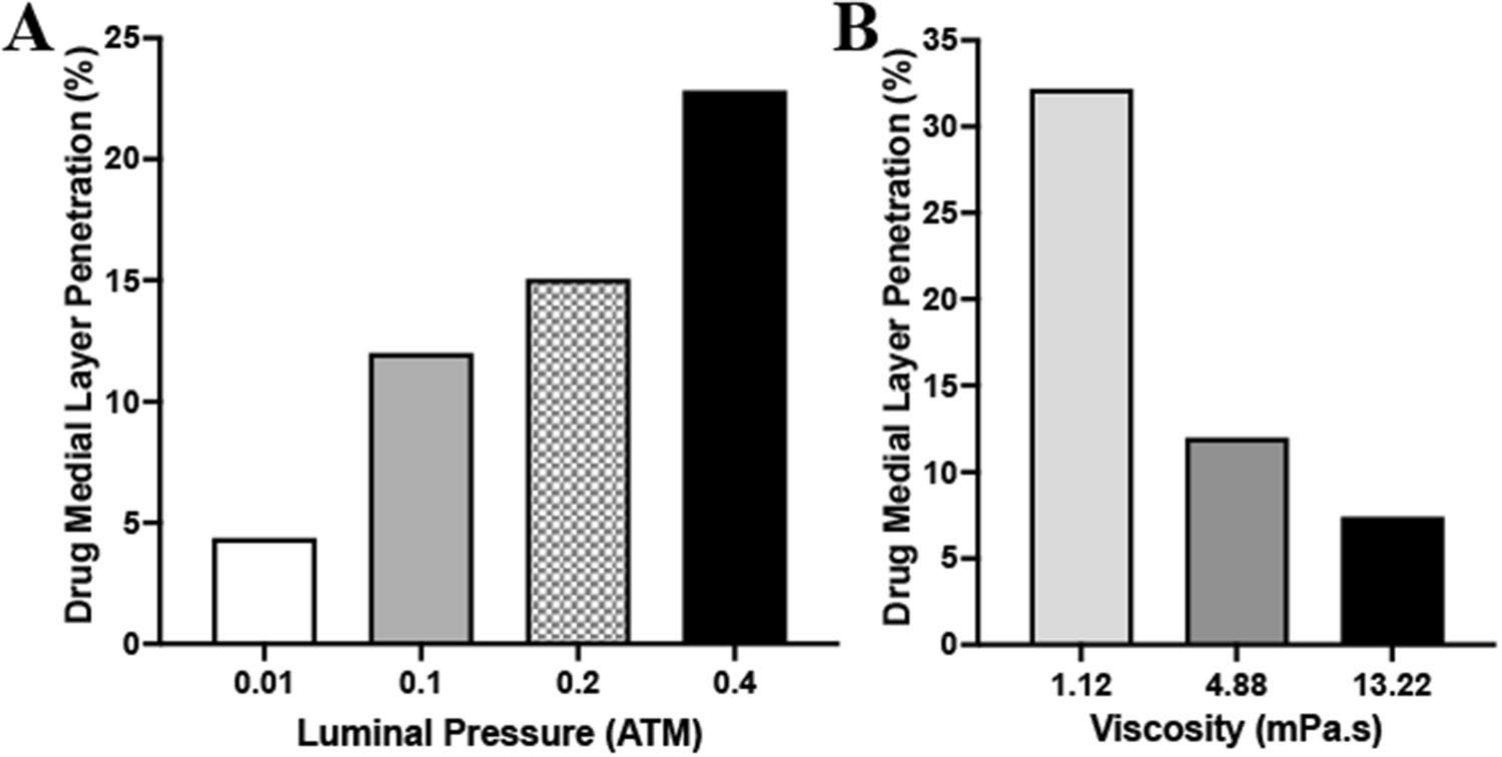
Mathematical results of varying pressures and viscosities. (**A**) Medial penetration depth of varying luminal pressures ranging from 0.01 to 0.4 atm. (**B**) Medial penetration depth of varying liquid viscosities ranging from 1.12 to 13.22 mPa s.

Mathematical results following 2 min of drug penetration into the medial layer demonstrated increasing drug penetration with increasing pressure conditions at the lumen-intima interface (Fig. 3). At 0.01 atm, medial penetration of the 4.88 mPa s fluid was measured at 4.37%. At 0.1, 0.2 atm and 0.4 atm, drug penetration was increased to 12.0%, 15.06% and 22.84%, respectively. To determine the impact of viscosity on liquid drug penetration, we again selected three different viscosity levels (1.12 mPa s, 4.88 mPa s, 13.22 mPa s) all delivered at a luminal pressure of 0.1 atm. The penetration level was greatest for the 1.12 mPa s fluid at 32.2% medial layer penetration. This level decreased to 12% and 7.42% for the 4.88 mPa s and 13.22 mPa s., respectively. Greater penetration of the 1.12 mPa s fluid could be attained by increasing pressures to 0.2 atm (38.3% medial penetration) and 0.4 atm (72.96% medial penetration).

### In vivo results

**Figure 4:**
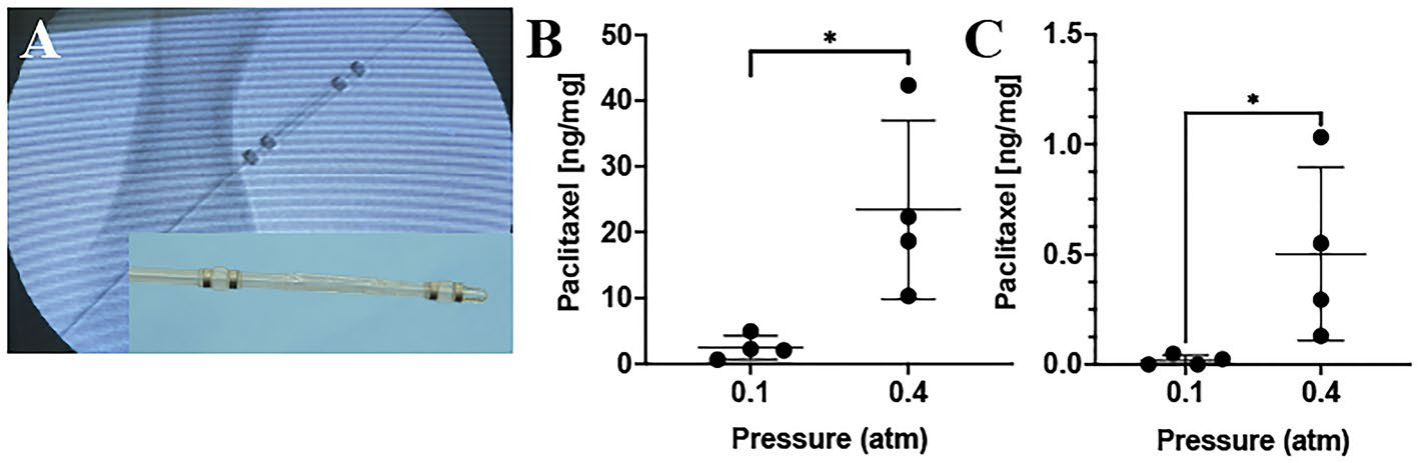
In vivo pharmacokinetic results of varying pressures. (**A**) Angiogram of the perfusion catheter during liquid drug delivery. The liquid paclitaxel is shown filling the treatment chamber (yellow arrows). The occlusion perfusion balloons, which create the treatment chamber, are shown in red arrows. The insert is a picture of the perfusion catheter. (**B**) Arterial paclitaxel retention at 1 h post-delivery for varying treatment chamber (luminal) pressures. (**C**) Arterial paclitaxel retention at 7 days post-delivery for varying treatment chamber (luminal) pressures. *P < 0.05, *n* = 4.

Following ex vivo and mathematical studies, in vivo studies were performed using the pig injury model to determine the impact of luminal (or treatment chamber) pressure on acute arterial paclitaxel retention. Arteries were treated with liquid paclitaxel at a pressure of 0.1 atm or 0.4 atm using the perfusion catheter. An angiogram of the perfusion catheter within the ilio-femoral artery is shown in Fig. 4A. Results demonstrated an increase in drug retention when delivered at a pressure of 0.4 atm compared to 0.1 atm (0.4 atm: 23.43 ± 13.59 ng/mg vs. 0.1 atm: 2.49 ± 1.81 ng/mg, p = 0.026) at 1 h (Fig. 4). At 7 days post-treatment, there was a significant decrease in drug retention for both the 0.1 atm treated arteries (1 h: 2.49 ± 1.81 ng/mg vs. 7 day: 0.018 ± 0.023 ng/mg, p = 0.034) and the 0.4 atm treated arteries (1 h: 23.43 ± 13.59 ng/mg vs. 7 day: 0.50 ± 0.39 ng/mg, p = 0.015) as compared to 1-h post-treatment. In comparison of the two treatment groups at 7-days post treatment, results demonstrated an increase in drug retention delivered at 0.4 atm pressure versus 0.1 atm (p = 0.0496).

## Discussion

This study was designed to evaluate, for the first time, the impact of luminal pressure on the penetration of liquid therapeutics into arterial vessel wall using a range of experiments. Our ex vivo studies, performed using harvested porcine carotid arteries, demonstrated significant increase of drug penetration at 0.1 and 0.4 atm. Furthermore, viscosity of the liquid therapy was shown to impact drug delivery into the vessel wall, with greater penetration at lower viscosity. A computational model, using a two-dimensional geometric model of the arterial wall, also demonstrated increasing drug penetration into the medial layer as pressure increased and viscosity was reduced. The culminating in vivo studies, using a pig peripheral injury model, demonstrated significant drug retention in arteries treated with a treatment chamber pressure of 0.4 atm versus 0.1 atm at both 1 h and 7 days. The overall results suggest higher luminal (or treatment chamber) pressure increases liquid drug penetration and retention into the arterial wall.

In this study, the varying luminal pressure ranged from 0 to 0.4 atm (0 to 304 mmHg). Although higher pressures could have been evaluated, luminal (or treatment chamber) pressures exceeding 0.4 atm (> 300 mmHg) are not clinically feasible and may result in the damage of the artery. For the in vivo studies, it is worth noting that the delivery pressure, defined by the pressure measured by the indeflator device, differed than the treatment chamber pressure (the pressure within the lumen of the treatment area). For the 0.1 atm and 0.4 atm groups, the average delivery pressure was measured at 6.38 ± 2.45 atm and 7.25 ± 1.49 atm, respectively. Our studies did not reveal any correlation between the indeflator pressure and the treatment chamber pressure. The target treatment chamber pressure (0.1 to 0.4 atm) was achieved at varying indeflator pressures ranging from 3.0 to 10.0 atm. The lack of correlation between the indeflator and treatment chamber pressures is most likely due to the target artery having compliance (not a rigid tube) and vessel branching. These results suggest the importance of measuring and monitoring the treatment chamber (luminal) pressure to ensure adequate liquid drug delivery while minimizing barotrauma and drug volume.

The in vivo results demonstrated a significant increase of acute drug retention at 1 h post-delivery in the 0.4 atm- versus the 0.1 atm-treated arteries at 1 h (0.4 atm: 23.43 ± 13.59 ng/mg vs. 0.1 atm: 2.49 ± 1.81 ng/mg, p = 0.026) and 7 days (0.4 atm: 0.50 ± 0.39 ng/mg vs. 0.1 atm: 0.018 ± 0.023 ng/mg, p = 0.0496) . However, it is worth noting that both the 0.1 atm and the 0.4 atm treated arteries showed significant decrease in paclitaxel tissue concentration from 1 h to 7 days. This decrease in arterial drug tissue concentration is most likely attributed to the choice of therapeutic drug. The paclitaxel utilized for this study was an oncological liquid paclitaxel, which are designed to be highly soluble. These drugs possess a half-life of approximately 6 h and their solubility allows for rapid cellular uptake^20,21^. However in DCBs, the strategy differs and the paclitaxel is designed to be insoluble using a mixture of crystalline and amorphous structures, increasing the half‐life to weeks to months^22–24^. The crystallinity increases long‐term arterial tissue paclitaxel residency, however any loss of the paclitaxel coating during transit and deployment can potentially remain and accumulate within distal tissue and organs. Literature has shown that roughly 1% to 10% of DCB paclitaxel coating gets transferred into the arterial wall, leaving the remaining (up to 90%) lost into the circulation^23,25^. DCB coating particulates has been shown to lead to fibrinoid necrosis within downstream skeletal muscle tissue^26,27^. Clinically, studies have also confirmed regional and temporal distribution of hypersensitivity and panniculitis secondary to particle embolization of commercial DCBs^28–30^.

**Figure 5:**
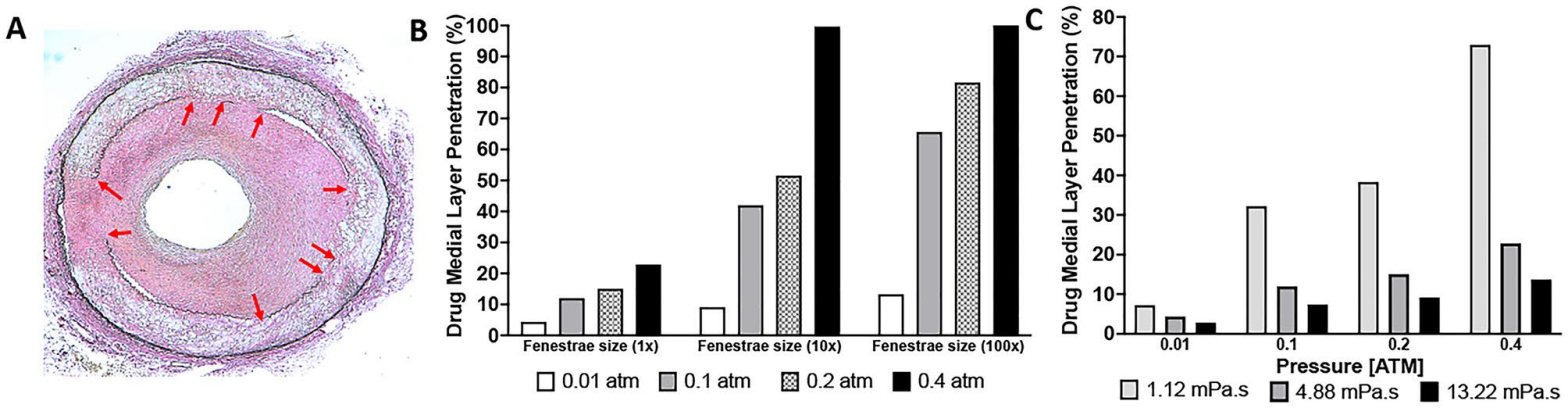
The impact of fenestrae size on liquid delivery. (**A**) Histological image of a disease artery showing disruption in the IEL (red arrows). (**B**) Mathematical results of varying fenestrae size at luminal pressures ranging from 0.01 atm to 0.4 atm. (**C**) Mathematical results of varying liquid viscosities at luminal pressures ranging from 0.01 atm to 0.4 atm.

The current study primarily focused on the impact of pressure on drug penetration, however, our mathematical model permits us to investigate other parameters. The fenestrae, which allows passage of drug into the medial layer, could also play an important factor in influencing drug penetration into the vessel wall. Our computational model was based on the fenestrae size associated with a healthy IEL, however, previous studies showed that arterial diseases such as intimal hyperplasia and atherosclerosis causes damage to the IEL and enlargement of fenestrae pores (Fig. 5A)^31,32^. Furthermore, vessel preparation prior to drug treatment, including debulking and plaque modifications using atherectomy devices and cutting balloons can result in further damage to the IEL and the fenestra. We thus investigated the impact of fenestra sizing on drug penetration. Specifically, we evaluated fenestra diameter sizes of 10-times and a 100-times larger than the healthy fenestra diameter for varying pressures. The computational data demonstrated an increase in drug penetration based on fenestrae size, with near complete penetration of the medial layer (> 99%) at 0.4 atm for both the 10-times and 100-times the common fenestrae pore sizes (Fig. 5B). At 0.1 atm, penetration increased to 41.9% for fenestra size 10-times the common fenestra size and further increased to 65.63% at 100-times the common fenestra size. Clinically, these data suggests drug penetration will be greater in damaged IEL and fenestrae. As only diseased arteries are treated in patients, along with pre-dilatation using balloon angioplasty and atherectomy, targeted arteries in a clinical setting should have increased fenestrate size, permitting medial drug penetration at pressure ranges between 0.1 to 0.4 atm.

Based on our ex vivo and mathematical results, drug viscosity is an additional factor that can impact the drugs medial penetration in a local liquid delivery approach. Specifically, we demonstrated a significant increase in drug penetration at a viscosity of 1.12 mPa s as compared to 4.88 mPa s. and 13.22 mPa s. (p < 0.0001) at a luminal pressure of 0.1 atm. The main viscosity modeled for this studies, 4.88 mPa s, was based on the manufacturers recommended dilution of the liquid paclitaxel, an oncological IV solution resulting in a paclitaxel concentration of 2.4 mg/ml. We additionally tested the impact of two other viscosities, 1.12 mPa s and 13.22 mPa s, representing a liquid paclitaxel concentration of 1.2 mg/ml and 3.6 mg/ml, respectively. Using the mathematical model, we further investigated the impact of viscosity for pressures ranging from 0.01 atm to 0.4 atm. The results of the impact of varying viscosities are shown in Fig. 5C. The simulation shows an inverse relationship between viscosity and medial penetration. Maximum drug penetration, in all cases, occurred when the viscosity was at minimum (1.12 mPa s). As viscosity increased, the rate of penetration decreased. Penetration rate was reduced 93% when viscosity was increased from 1.12 mPa s to 4.88 mPa s. There was a further reduction in penetration (116%) when viscosity was increased to 13.22 mPa s.

The results from the ex vivo and computational models add clarity regarding the varying parameters associated with liquid drug penetration into the vessel wall, however there are some limitations to this study. The ex vivo studies used healthy arterial segments. The computational analysis was also performed using a non-endothelialized healthy arterial model with no disease. Human lesions are more complex and often include complications such as fibrosis, necrotic cores, hemorrhage and calcification. In addition, the interaction between the drug and tissue, drug binding potential, drug diffusivity and drug accumulation in the arterial wall were not considered^33^. Heterogeneity due to endothelial denudation, disease and uneven atherosclerotic lesions can potentially affect drug penetration into the arterial wall, resulting in uneven distribution and penetration^34^. Regardless, non-diseased healthy arteries are the current standard models to determine the pharmacokinetics of intervascular devices.

In conclusion, this study demonstrated the importance of pressure in the local delivery of liquid therapy into the arterial wall. All three models, ex vivo, mathematical and in vivo*,* demonstrated higher delivery pressure leads to greater drug penetration into the medial layer. Our results indicated medial drug penetration can be achieved at luminal pressures ranging from 0.1 to 0.4 atm. Additional studies are warranted to further evaluate the impact of varying pressure on mid- to long-term drug retention and the accompanied biological outcomes.

## Materials and methods

### Ex vivo model

Porcine carotid arteries were harvested from large pigs (110–160 kg) from a local abattoir and transferred in sterile Phosphate Buffered Saline (PBS) with 1% antibiotics-antimycotic (Gibco, Grand Island, NY, 14072). Vessels were then rinsed in sterile PBS in a culture hood. The excess fat, connective tissue, and fascia were removed from each vessel. Vessels were cut into approximately 8 cm segments and placed into the custom-made bioreactor housing apparatus. The ex vivo arteries then underwent endothelial denudation using an angioplasty balloon catheter (5.0 mm × 15 mm). The bioreactor was kept inside the incubator for at least 30 min prior to testing to obtain a temperature of 37 °C.

To fill the artery with liquid therapy, the bioreactor system was first placed vertically and a fiberoptic pressure sensor was placed inside the artery to monitor the luminal pressure applied by the drug delivery. The vertical position allows complete filling of luminal space without minimal pressure. The fluorescently labeled paclitaxel drug (0.1 mg/ml, Flutax-1, Tocris Bioscience, Bristol, UK) was delivered slowly via an indeflator device (Abbott Vascular) through the bottom port of the bioreactor while the top port was open to minimize luminal pressure within the artery during this process. Once the artery was completely filled, the top valve was closed. The indeflator device was then used to gradually increase the luminal pressure within the artery ranging from 0 to 0.4 atm for 2 min. The luminal pressure was monitored and recorded via a computer. Following the procedure, the artery was flushed out with 50 ml of saline, cut into 1–2 cm segments and processed for frozen sectioning. A schematic illustration of the ex vivo setup is shown in Fig. 1. For the ex vivo studies, three Flutax-1 liquid solutions with viscosities of 1.12 mPa s, 4.8 mPa s and 13.22 mPa s were tested. The varying Flutax-1 viscosities were accomplished using a mixture of saline and purified polyoxyl 35 castor oil and dehydrated alcohol (Sagent Pharmaceuticals, Schaumburg, IL) at mixing ratio of 0.1:4.9 (castor oil/dehydrated alcohol:saline) for 1.12 mPa s, a ratio of 1:4 for 4.88 mPa s and a ratio of 3:2 for 13.22 mPa s.

### Arterial sectioning and imaging

To determine drug penetration, the cut arterial segments were frozen fresh in an optical cutting temperature (O.C.T.) compound (Sakura Finetek USA, Inc., Torrance, CA, USA). Arterial cross-sections were sectioned at a thickness of 12 microns using cryosection and imaged with a Nikon Eclipse TI confocal microscope (Nikon Instruments Inc., Melville, NY, USA). Fluorescently labeled paclitaxel (Flutax-1) was visualized at an excitation wavelength of 488 nm and detection at 525/50 nm. To measure the depth of drug penetration, confocal images were analyzed using ImageJ software (NIH). Specifically, the length of penetration for each section was measured from the IEL to maximum penetration depth and normalized to the thickness of the medial layer or wall thickness.

### Geometric model

**Figure 6:**
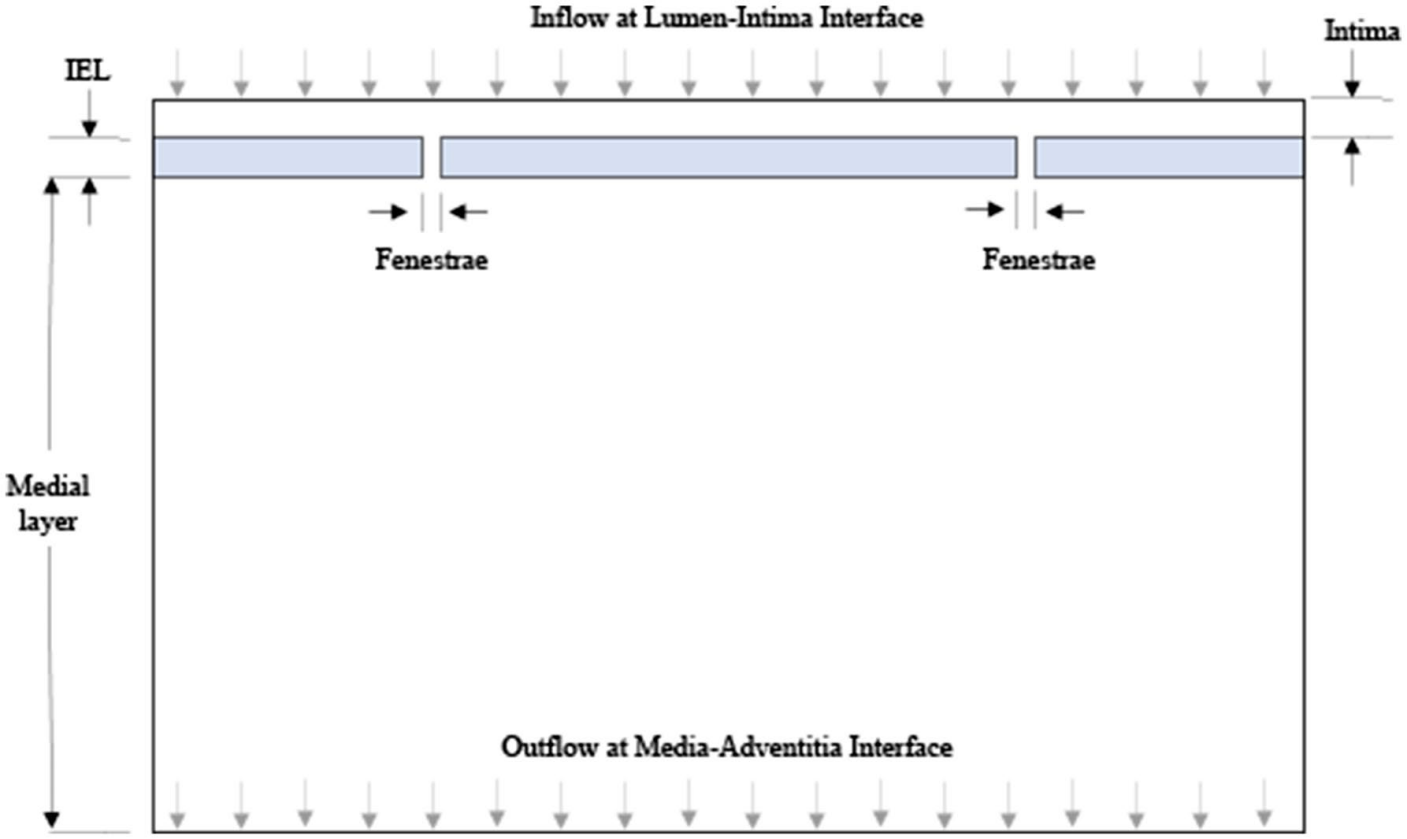
Schematic drawing of the two-dimensional geometric model implemented in this study.

A simplified two-dimensional geometric model of the arterial wall was developed for this study. Three layers of the artery were modeled, including the intima (the innermost layer), the internal elastic lamina (modeled as an impermeable membrane consisting of fenestrae), and the medial layer (Fig. 6). The passage of fluid from the intimal into the medial layer is via cylindrical pores called fenestrae^35^. The endothelial layer, which is present between the lumen and intima, was excluded in this study since treated arteries typically lack endothelial cells. The adventitia layer was also excluded as the focus of the study was to quantify liquid drug penetration from the intima into the medial layer.

### Boundary conditions and material properties

To account for the changes in the material properties of the multilayer model, specifically taking into account the influence of higher luminal pressure on the intimal and medial layers, four distinct models were utilized (Table 1). The pressure dependent variables for each pressure-dependent model included the fenestrae diameter, permeability and porosity, similar to previous idealized geometries to model transmural flow through the vessel wall^35^. The fenestrae diameter, permeability and porosity values at 0.1 atm and 0.2 atm were based on previous study^35^. The remaining values for the fenestrae diameter, permeability and porosity for 0.01 atm and 0.4 atm were linearly extrapolated. The intima and fenestrae are composed of a porous collagen fibers in a proteoglycan matrix. In this model, the intima, fenestrae, and media are considered to have uniform material properties (permeability and porosity) throughout^35^.

**Table 1:** Summary of pressure-dependent geometric and material properties.

Pressure-dependent models	Luminal delivery pressure [atm]	Fenestrae diameter [μm]	Permeability [m^2^]	Porosity
1	0.01	0.0359	4.45 × 10^–17^	0.00297
2	0.1	0.0834	4.01 × 10^–17^	0.7947
3	0.2	0.089	1.58 × 10^–17^	0.6378
4	0.4	0.101	6.24 × 10^–18^	0.4448

### Computations method

The Finite Element Analysis Method (Ansys Fluent, Version 16.2) was used to analyze the data. The distance between fenestrae was set at 25 μm based on the approach used and assumptions made by Dabagh and colleagues^36^. The thickness of the internal elastic lamina was determined to be 1 μm^36^. The density and the dynamic viscosity of blood plasma were considered to be 1000 kg/m^3^ and 0.72 mPa s, respectively^36^. The liquid drug solution viscosities tested were 1.12 mPa s, 4.88 mPa s and 13.22 mPa s. A computational mesh was generated over the domain to approximate a geometric property. In this study, flow was considered to be laminar for all cases. The initial conditions at zero seconds assumed that 100% of the drug mixture volume entered at the inlet and the initial volume of the artery contained only blood plasma. This 2D FE parametric model will thus help to investigate and better understand the effects of different parameters such as arterial wall porosity, drug viscosity, fenestration size and distribution, arterial wall thickness and luminal delivery pressure.

### Pig injury model

This study was approved by the University of South Alabama Institutional Animal Care and Use Committee (IACUC) and conformed to the current Guide for the Care and Use of Laboratory Animals and in compliance with the ARRIVE guidelines. The experimental preparation of the animal model has been previously reported^37,38^. Four female pigs (12.3–14.1 kg) were pre-medicated with ketamine and midazolam and intubated and placed on a ventilator. While the animals were ventilated on isoflurane gas anesthesia. the right carotid artery was exposed under a sterile field. The caudal end of the right carotid artery was tied-off. Using micro scissors, a small incision was made to the right carotid artery and a 6 French (F) guide sheath was inserted. A NITREX 0.014 guidewire (ev3 Inc., Plymouth, MN) was inserted and, under fluoroscopic guidance, endothelial denudation using a 4 × 12 mm angioplasty balloon catheter (Abbot Vascular, Abbott Park, IL) was performed to the left and right iliac arteries. Following denudation, liquid paclitaxel (Sagent Pharmaceuticals, Schaumburg, IL) was delivered via a perfusion catheter (3.0 mm × 30 mm, Advanced Catheter Therapies, Chattanooga, TN) at a luminal delivery pressure of 0.1 or 0.4 atm for two minutes. The perfusion catheter has two compliant occlusion balloons (one proximal and one distal) which define the treatment chamber. Luminal pressure, or treatment chamber pressure, was measured real-time via a sensor located within the treatment chamber which was connected to an external pressure monitor for the purpose of monitoring the pressure inside the treatment chamber during infusion of the liquid drug. Antiplatelet therapy consisted of aspirin (40 mg/day) given orally 24 h before catheterization, while single-dose intra-arterial heparin (150 IU/kg) and lidocaine administered at the time of catheterization. One hour and seven days after treatment, anesthetized animals were euthanized and the treated segments removed based on landmarks identified by angiography. Excised arteries were analyzed for tissue drug concentrations using a validated, previously described high-performance liquid chromatography (HPLC)-electrospray ionization- tandem mass spectrometry system (LC–MS/MS)^38–41^.

### Statistical analysis

All values were expressed as mean ± standard deviation (SD). Continuous variables were compared between groups using one‐way analysis of variance (ANOVA) using GraphPad Prism 9 (GraphPad Software, La Jolla, CA, USA). A value of p ≤ 0.05 was considered statistically significant. If statistical significance was shown, comparison of quantitative data of multiple groups was performed by Tukey’s multiple comparisons post hoc test.
